# A Positive Depression Screen Is Associated with Emergency Medicine Resident Burnout and Is not Affected by the Implementation of a Wellness Curriculum

**DOI:** 10.5811/westjem.2021.9.52016

**Published:** 2021-10-26

**Authors:** Kelly Williamson, Patrick M. Lank, Adriana Olson, Navneet Cheema, Elise Lovell

**Affiliations:** * Northwestern University, Feinberg School of Medicine, Department of Emergency Medicine, Chicago, Illinois; † University of Chicago, Pritzker School of Medicine, Department of Emergency Medicine, Chicago, Illinois; ‡ University of Illinois at Chicago, Advocate Christ Medical Center, Department of Emergency Medicine, Chicago, Illinois

## Abstract

**Introduction:**

While burnout is occupation-specific, depression affects individuals comprehensively. Research on interventions for depression in emergency medicine (EM) residents is limited.

**Objectives:**

We sought to obtain longitudinal data on positive depression screens in EM residents, assess their association with burnout, and determine whether implementation of a wellness curriculum affected the rate of positive screens.

**Methods:**

In February 2017, we administered the Maslach Burnout Inventory and the Primary Care Evaluation of Mental Disorders Patient Health Questionnaire two-question depression screen at 10 EM residencies. At five intervention sites, a year-long wellness curriculum was then introduced while five control sites agreed not to introduce new wellness initiatives during the study period. Study instruments were re-administered in August 2017 and February 2018.

**Results:**

Of 382 residents, 285 participated in February 2017; 40% screened positive for depression. In August 2017, 247/386 residents participated; 27.9% screened positive for depression. In February 2018, 228/386 residents participated; 36.2% screened positive. A positive depression screen was associated with higher burnout. There were similar rates of positive screens at the intervention and control sites.

**Conclusion:**

Rates of positive depression screens in EM residents ranged between 27.9% and 40%. Residents with a positive screen reported higher levels of burnout. Rates of a positive screen were unaffected by introduction of a wellness curriculum.

## INTRODUCTION

Burnout and depression are important and challenging issues facing resident physicians today. When first identified, burnout was thought to result from negative work-life balance, mental and physical exhaustion, and job disengagement and dissatisfaction.^1^ Today burnout is defined as the triad of emotional exhaustion, depersonalization, and low personal achievement, thought to result from system pressures and an imbalance between overwhelming job demands and insufficient job resources and support.^2^ Physician burnout is recognized as a widespread phenomenon affecting over half of practicing physicians. Emergency physicians report burnout levels between 55–70%.^3^–^6^ Additionally, resident physicians and fellows have higher levels of burnout and are more likely to screen positive for depression than population controls.^7^ Several studies have highlighted the association of burnout with mental health consequences, alcohol and substance abuse disorders, and suicidal ideation.

A survey of anesthesiologists determined that there was an independent association between lower mental and physical composite health scores and greater burnout scores.^8^ Physicians across all specialties have rates of alcohol abuse and dependence higher than the general population and these are independently associated with burnout.^9^ In particular, emergency physicians and emergency medicine (EM) residents experience higher rates of substance abuse than other specialties, and an estimated 4.9–12.5% of EM residents consume alcohol on a daily basis.^10^,^11^ In a study of nearly 8,000 surgeons, 1 in 16 reported suicidal ideation in the prior year and each one point increase in burnout scores correlated with an increased likelihood of reporting suicidal ideation.^12^ These studies suggest that burnout is a complex syndrome that does not exist in isolation but rather is intimately connected to overall physician well-being.

Given the prevalence of burnout and its established detrimental effects, the Accreditation Council for Graduate Medical Education (ACGME) introduced a new Common Program Requirement, effective in 2017, that mandates resident education on the identification of burnout, depression, and substance abuse, and the implementation of program efforts to encourage well-being.^13^ There are graduate medical education programs proactively addressing mental health issues and depression in residents. The Pediatric Integrative Medicine in Residency program introduced a 100-hour online educational curriculum into five pediatric residencies.^14^ At the Oregon Health and Science University, a resident and faculty wellness program that provides educational outreach, psychological counseling, and psychiatric evaluation was associated with high participant satisfaction and a 10-year growth in utilization of the offered services.^15^ At Cooper University, group meetings with an employee assistance program (EAP) counselor were scheduled for all interns within the first six months of their residency, with the objectives of destigmatizing use of the EAP and increasing familiarization with the services. The initial seven sessions were so well received that three additional sessions were added and utilization thereafter significantly increased.^16^

Yet studies on wellness interventions in EM residents have been limited. We therefore sought to determine the prevalence of positive depression screens in EM residents over one year, the association of positive depression screens with burnout, and whether the implementation of a wellness curriculum affected the rate of positive depression screens in EM residents.

## METHODS

### Study Design

This study was part of a larger, multicenter prospective educational trial performed at 10 ACGME-accredited EM residencies in the United States.^17^ Members of the Emergency Medicine Education Research Alliance (EMERA) were core faculty at all sites at the time of study initiation. The study was reviewed by each institution’s institutional review board and received approval at each site prior to study initiation.

**Population Health Research Capsule**

What do we already know about this issue?

*Burnout and depression are challenging issues facing emergency medicine residents, but research is sparse.*

What was the research question?

*What is the prevalence of positive depression screens in residents and their association with burnout? Do wellness curricula affect the rate of positive depression screens?*

What was the major finding of the study?

*Rates of positive depression screens were between 27.9–40%, were associated with higher rates of burnout, and these rates were unaffected by a wellness curriculum.*

How does this improve population health?

*In determining wellness best practices for residents, detecting depression and promoting mental health resources are critically important.*

### Subjects

Eligible subjects for this study were postgraduate year (PGY) 1–4 EM residents at the participating programs during the study period February 2017–February 2018. Surveys were administered to current residents at each program. Participation in the survey study was voluntary. Informed consent was obtained from all subjects.

### Study Protocol

#### Survey Instrument

The survey instrument was sent to eligible participants at all study sites at three different time points: February 2017; August 2017; and February 2018. The survey was administered either as a paper survey or via online, proprietary software SurveyMonkey (Momentive, Inc, San Mateo, CA) at the preference of the site study leader. Follow-up for nonresponders was program-specific, either in person or via email. The survey instrument was designed for completion in 15 minutes and consisted of a total of 34 questions. In addition to questions related to demographic information, the instrument consisted of several tools established for use in physician wellness research.

The Maslach Burnout Inventory (MBI) is considered the gold standard in the assessment of physician burnout, measuring the domains of emotional exhaustion, depersonalization, and personal accomplishment.^18^ The survey instrument also included the Primary Care Evaluation of Mental Disorders Patient Health Questionnaire two-question screen (PRIME-MD PHQ-2)^19^ and three additional published wellness instruments: a quality of life assessment; an appraisal of career satisfaction; and a work-life balance rating.^20^–^22^ The PRIME-MD PHQ-2 depression screen asks the following questions: “During the past month, have you often been bothered by feeling down, depressed, or hopeless?”; and “During the past month, have you often been bothered by little interest or pleasure in doing things?” A “yes” response to either question is considered a positive screen. In a validation study, the Prime-MD PHQ-2 performed similarly to longer survey tools, including the long and short forms of the Center for Epidemiologic Studies Depression Scale, the long and short forms of the Beck Depression Inventory, the Symptom-Driven Diagnostic System for Primary Care, the Medical Outcomes Study depression measure, and the Quick Diagnostic Interview Schedule. In addition, a positive response on the two-item instrument had a sensitivity of 96% and a specificity of 57% for detecting depression when compared with clinical interviews.^19^

#### Curriculum Intervention

Prior to the first survey administration, each site self-selected into the control or intervention group based on available site resources to institute the wellness curriculum. A year-long. multifaceted wellness curriculum was introduced at the five intervention sites in March 2017, while the five control sites agreed not to introduce new wellness initiatives during the study period. Individual participation in all elements of the curriculum was highly encouraged but not mandated. No incentives were provided for participation in the curriculum. Complete details of the wellness curriculum, as well as resident participation and perceptions have been previously published.^23^,^24^ The comprehensive curriculum included standardized, structured didactics presented by the study investigator at each site every other month, individualized interactive instruction assignments, additional reading materials and resources, and internet-based opportunities.^23^ The curricular intervention was completed prior to administration of the February 2018 end-of-study survey.

#### Analysis

In addition to the MBI and the Prime-MD PHQ-2, we obtained basic demographic information that included respondent age, gender, ethnicity, and PGY classification. Results of the components of the MBI are presented as both continuous and dichotomous data. “Global burnout” was defined as having both an emotional exhaustion score >26 and a depersonalization score >12 at any single survey administration.^18^,^25^

Descriptive statistics are presented as total number (n) and percentages with 95% confidence intervals for categorical variables. Continuous variables are displayed as either means with standard deviation for normally distributed variables or as medians with interquartile ranges (IQR) for non-normally distributed variables. Univariate analyses were performed using chi-square or Student’s t-test, as appropriate, for continuous or categorical variables. We performed logistic regression to obtain adjusted odds ratios for burnout at each survey administration for intervention and control site respondents. Analysis was performed using a statistical package program R version 3.3.2 (R Foundation for Statistical Computing, Vienna, Austria).

## RESULTS

A total of 285/382 (74.6%) residents participated in the February 2017 data collection; 40% screened positive for depression. In August 2017, 247/386 (64%) residents participated; 27.9% screened positive. In February 2018, 228/386 (59%) residents participated; 36.2% screened positive. There were no significant differences in age, gender, ethnicity, or PGY training year distribution between the control and the intervention sites (Table 1). There were no statistical differences in the rates of positive depression screens between the intervention and control sites at any of the three data collections or over time. In addition, there was no sustainable change in positive depression screens within the intervention group during the study period (Table 2).

We assessed the three components of burnout as continuous variables and compared the means for each component score with the results of the depression screen. Residents who screened positive for depression experienced higher emotional exhaustion (mean 26.8 in screen-positive population vs 18.0 in screen-negative population, *P* <0.0001), higher depersonalization (mean 15.2 in screen positive vs 11.3 in screen negative, *P* <0.0001), and lower personal accomplishment (mean 36.3 in screen positive vs 40.9 in screen negative, *P* <0.0001) (Table 3).

Consistent with Maslach’s definition, global burnout was defined as having both an emotional exhaustion score > 26 and a depersonalization score > 12.^18^,^25^ Positive depression screens were significantly associated with global burnout in our study population. At each survey administration, residents who screened positive for depression were significantly more likely to meet criteria for burnout (all *P* <0.005) (Table 4). This association remained significant when controlling for the potential confounders of the wellness curriculum intervention and respondent demographics (age, gender, ethnicity, and PGY status). In addition, when controlling for age, gender, ethnicity, and PGY status using logistic analysis, those meeting criteria for burnout among respondents who screened positive for depression was significant at each survey administration (Table 5).

## DISCUSSION

In this year-long national study of EM residents, the prevalence of positive depression screens as measured by the PRIME-MD PHQ-2 was 27.9–43%. A positive depression screen was significantly associated with both global burnout as well as the individual components of burnout. The rates of positive depression screens were unaffected by the introduction of a multifaceted wellness curriculum. This study represents the first EM multi-center educational intervention trial to assess the effects of implementation of a formalized wellness curriculum on EM resident depression screens.

The prevalence of a positive depression screen in our survey sample ranged from 27.9–43%, higher than the 12% prevalence previously reported in a single-center study of EM residents.^26^ A systematic review and meta-analysis determined a 28.8% pooled prevalence of depression or depressive symptoms in resident physicians.^27^ The higher rates of a positive depression screen in our study population may relate to different measurement methods, an increasing prevalence of depression symptoms in resident physicians, or a higher rate of depression symptoms in EM residents compared with residents of other specialties.

There has recently been debate regarding the relationship between physician burnout and depression. The association between burnout and positive depression screens is well described.^6^,^28^,^29^ Physicians experiencing burnout are also more likely to suffer from major depression.^30^ Some proponents advocate for the classification of burnout as a depressive condition given its association with the depressive symptoms of dysphoria, anhedonia, and exhaustion, and posit that the components of burnout correlate more highly with depression than with each other.^31^ However, others support the concept that depression is a disease that has well-defined diagnostic criteria and is context-free, while burnout is a separate, job-related syndrome that is situation-specific.^32^ A recent systematic review also supports depression, anxiety. and burnout as being distinct and robust constructs.^33^

Several factors may have contributed to the lack of effect of the formalized wellness curricula on rates of positive depression screens. During the study period, there was an increased awareness and promotion of physician wellness on a national level. This includes the Council of Emergency Medicine Residency Directors (CORD)/American College of Emergency Physicians National Physician Suicide Awareness Day campaign, the Academic Life in Emergency Medicine Wellness Think Tank, and the CORD mini-fellowship in wellness leadership. While the control sites agreed not to introduce any new programmatic wellness initiatives during the study period, residents may have been exposed to burnout and mental health initiatives at an institutional and national level, thereby accessing broader wellness initiatives despite not engaging in the study curriculum at their program. In addition, within the curriculum, mental health was addressed in the physical and emotional sections, but this was not a specific mental health curriculum. Finally, while the curriculum was highly encouraged, participation was not mandatory and there was variable compliance.^24^

## LIMITATIONS

There are several important limitations to our study. We used a convenience sample of residents, which was not subject to power analysis. As the analysis compared the intervention sites with the control sites, we did not account for or follow which particular residents were involved in each survey analysis. It is possible that bias was introduced by having different respondents during the different survey administrations. There may also have been a selection bias with regard to depressive symptoms among the residents choosing to complete the surveys. Additionally, individual sites self-selected into the intervention and control groups based on available resources, which may have introduced selection bias.

As the control sites did not have the resources to implement the multifaceted wellness curriculum, it is also possible that there was less programmatic support to promote a new wellness culture at the time or that the control sites may have been satisfied with the wellness interventions already in place at their programs. To that extent, while the control sites agreed not to introduce new wellness initiatives during the study period, they may have already had formal or informal wellness activities and mental health resources in place that affected the results of the study. In addition, residents within the control sites programs may still have independently accessed national wellness resources that were becoming increasingly prevalent during the study period.

We chose not to use a hierarchical model to control for nesting by residency programs in each of the two groups but rather a priori to treat them as a larger group of control vs intervention sites. When we performed statistical analysis, using both logistic regression and mixed effects, participant site itself was not a confounding variable at any point in the study. There is also likely a seasonal variation with respect to wellness, which may be especially notable in certain geographic areas. Two of our data collections were conducted in February, close to the annual EM in-training examination and in the middle of winter, which may have negatively affected wellness and mental health during those two survey administrations. Also, while the PRIME-MD PHQ-2 is a sensitive screen for detecting depression, the specificity of 57% is quite low and may have led to an overestimate of prevalence. Finally, we did not account for residents’ rotations during the time of each survey administration.

## CONCLUSION

In this one-year study, rates of positive depression screens in EM residents ranged between 27.9% and 40%. Emergency medicine residents with a positive depression screen also reported higher levels of burnout, and the rates of a positive screen were unaffected by the introduction of a wellness curriculum. As residencies seek to determine wellness best practices, attention to depression detection and independent promotion of mental health resources are critically important.

## Supplementary Information



## Figures and Tables

**Table 1:** Demographics of emergency medicine residents who responded to survey.

Variable	Control	Intervention
Age	29 (IQR: 28–32)	29 (IQR: 27–31)
Gender (% female)	35.3% (95% CI, 28.1–42.5%)	29.1% (95% CI, 22.4–35.7%)
Ethnicity (% under-represented in medicine)	10.3% (95% CI, 5.4–15.3%)	6.4% (95% CI, 2.4–10.5%)
Postgraduate year		
PGY 1	42	41
PGY 2	48	45
PGY 3	44	44
PGY 4	0	10

*IQR*, interquartile range; *CI*, confidence interval; *PGY*, postgraduate year.

**Table 2:** Percentage positive depression screens.

	Control	Intervention	*P* value
February 2017	43%	36.9%	0.35
August 2017	32.2%	21.8%	0.09
February 2018	32.6%	41.4%	0.22

**Table 3:** Mean burnout scores and depression screen results.

	Depression Screen Positive	Depression Screen Negative	*P* value
Emotional Exhaustion	26.8	18	<0.0001
Depersonalization	15.2	11.3	<0.0001
Personal Accomplishment	36.3	40.9	<0.0001

**Table 4:** Global burnout and depression screens.

	Burnout Negative	Burnout Positive
Survey #1		
Depression Screen Negative	141 (53%)	20 (7%)
Depression Screen Positive	67 (25%)	39 (15%)
Survey #2		
Depression Screen Negative	156 (64%)	19 (8%)
Depression Screen Positive	42 (17%)	27 (11%)
Survey #3		
Depression Screen Negative	121 (54%)	22 (10%)
Depression Screen Positive	48 (21%)	33 (15%)

**Table 5:** Adjusted odds of meeting criteria for burnout among respondents who screen positive for depression†.

	Adjusted OR	*P* value
Survey 1	5.4 (2.8–10.1)	<0.001
Survey 2	4.3 (1.9–10.5)	<0.001
Survey 3	3.4 (1.5–8.2)	<0.005

† When controlling for age, gender, and ethnicity.

*OR*, odds ratio.
