# Supplementary material for: A Positive Depression Screen Is Associated with Emergency Medicine Resident Burnout and Is not Affected by the Implementation of a Wellness Curriculum
Source: West J Emerg Med. 2021 Oct 26;22(6):1341–6. doi: 10.5811/westjem.2021.9.52016 (PMC8597694; doi:10.5811/westjem.2021.9.52016)
Supplement: Supplementary file 1 [file wjem-22-1341-s001.docx]

Appendix 1

> fit5<-glm(well$burnout1~factor(well$Intervention)+well$Age_final+factor(well$Gender_all)+factor(well$ethurm)+factor(well$dep1), data=well, family=binomial())

> summary(fit5)

Coefficients:

                              Estimate Std. Error z value Pr(>|z|)

(Intercept)                -17.319578 882.744912  -0.020    0.984

factor(well$Intervention)2  -0.622185   0.354194  -1.757    0.079 .

well$Age_final               0.056351   0.055161    1.022    0.307

factor(well$Gender_all)1    13.701034 882.743429   0.016    0.988

factor(well$Gender_all)2    13.893811 882.743462   0.016    0.987

factor(well$ethurm)1         0.002534   0.617999   0.004    0.997

factor(well$dep1)1           1.685159   0.342283   4.923 8.51e-07 ***

> fit6<-glm(well$burnout2~factor(well$Intervention)+well$Age_final+factor(well$Gender_all)+factor(well$ethurm)+factor(well$dep2), data=well, family=binomial())

> summary(fit6)

Coefficients:

                           Estimate Std. Error z value Pr(>|z|)

(Intercept)                -3.82112    2.03039  -1.882 0.059841 .

factor(well$Intervention)2 -0.23144    0.43906  -0.527 0.598115

well$Age_final              0.08207    0.06882   1.192 0.233066

factor(well$Gender_all)2   -0.05451    0.48689  -0.112 0.910867

factor(well$ethurm)1        1.28303    0.76526   1.677 0.093619 .

factor(well$dep2)1          1.46702    0.43915   3.341 0.000836 ***

> fit7<-glm(well$burnout3~factor(well$Intervention)+well$Age_final+factor(well$Gender_all)+factor(well$ethurm)+factor(well$dep3), data=well, family=binomial())

> summary(fit7)

Coefficients:

                           Estimate Std. Error z value Pr(>|z|)

(Intercept)                -1.85530    1.98281  -0.936  0.34943

factor(well$Intervention)2  0.03870    0.44521   0.087  0.93073

well$Age_final              0.01892    0.06930   0.273  0.78487

factor(well$Gender_all)2    0.30229    0.44060   0.686  0.49266

factor(well$ethurm)1       -0.90497    1.13063  -0.800  0.42348

factor(well$dep3)1          1.23022    0.43428   2.833  0.00461 **

Table 6: Beta coefficients (with p-value) for logistic regression analysis at Survey 1.

|  | Beta coefficient | p-value | Adjusted OR |
| --- | --- | --- | --- |
| **Depression screen positive** | **1.6852** | **<0.005** | **5.39** |
| Underrepresented in medicine | 0.0025 | 0.997 | 1.00 |
| Age | 0.0564 | 0.307 | 1.06 |
| Gender (Female) | 0.1370 | 0.988 | 1.15 |
| Intervention site | -0.6222 | 0.079 | 0.54 |

Table 7: Beta coefficients (with p-value) for logistic regression analysis at Survey 2

|  | Beta coefficient | p-value | Adjusted OR |
| --- | --- | --- | --- |
| **Depression screen positive** | **1.4670** | **<0.005** | **4.33** |
| Underrepresented in medicine | 1.2830 | 0.094 | 3.61 |
| Age | 0.0821 | 0.233 | 1.09 |
| Gender (Female) | -0.05451 | 0.911 | 0.95 |
| Intervention site | -0.23144 | 0.598 | 0.79 |

Table 8: Beta coefficients (with p-value) for logistic regression analysis at Survey 3

|  | Beta coefficient | p-value | Adjusted OR |
| --- | --- | --- | --- |
| **Depression screen positive** | **1.2302** | **<0.005** | **3.42** |
| Underrepresented in medicine | -0.9050 | 0.423 | 0.40 |
| Age | 0.0189 | 0.785 | 1.02 |
| Gender (Female) | 0.3023 | 0.493 | 1.35 |
| Intervention site | 0.0387 | 0.931 | 1.04 |
